# Positive spill-over effects of ART scale up on wider health systems development: evidence from Ethiopia and Malawi

**DOI:** 10.1186/1758-2652-14-S1-S3

**Published:** 2011-07-06

**Authors:** Freya Rasschaert, Marjan Pirard, Mit P Philips, Rifat Atun, Edwin Wouters, Yibeltal Assefa, Bart Criel, Erik J Schouten, Wim Van Damme

**Affiliations:** 1 Institute of Tropical Medicine, Department of Public Health, Belgium, Antwerp, Belgium; 2 Imperial College London & The Global Fund to Fight AIDS, Tuberculosis and Malaria, Geneva, Switzerland; 3 Research Centre for Longitudinal and Life Course Studies, University of Antwerp, Belgium. & Centre for Health Systems Research & Development, University of the Free State, South Africa; 4 Medical Services Directorate, Federal Ministry of Health, Addis Ababa, Ethiopia; 5 Management Sciences for Health, Lilongwe, Malawi

## Abstract

**Background:**

Global health initiatives have enabled the scale up of antiretroviral treatment (ART) over recent years. The impact of HIV-specific funds and programmes on non-HIV-related health services and health systems in genera has been debated extensively. Drawing on evidence from Malawi and Ethiopia, this article analyses the effects of ART scale-up interventions on human resources policies, service delivery and general health outcomes, and explores how synergies can be maximized.

**Methods:**

Data from Malawi and Ethiopia were compiled between 2004 and 2009 and between 2005 and 2009, respectively. We developed a conceptual health systems framework for the analysis. We used the major changes in human resources policies as an entry point to explore the wider health systems changes.

**Results:**

In both countries, the need for an HIV response triggered an overhaul of human resources policies. As a result, the health workforce at health facility and community level was reinforced. The impact of this human resources trend was felt beyond the scale up of ART services; it also contributed to an overall increase in functional health facilities providing curative, mother and child health, and ART services. In addition to a significant increase in ART coverage, we observed a remarkable rise in user rates of non-HIV health services and an improvement in overall health outcomes.

**Conclusions:**

Interventions aimed at the expansion of ART services and improvement of long-term retention of patients in ART care can have positive spill-over effects on the health system. The responses of Malawi and Ethiopia to their human resources crises was exceptional in many respects, and some of the lessons learnt can be useful in other contexts. The case studies show the feasibility of obtaining improved health outcomes beyond HIV through scaled-up ART interventions when these are part of a long-term, system-wide health plan supported by all decision makers and funders.

## Background

Over recent years, antiretroviral treatment (ART) coverage has increased significantly thanks to the support of global health initiatives, such as the Global Fund to Fight AIDS, Tuberculosis and Malaria (Global Fund), and the US President’s Emergency Plan For AIDS Relief (PEPFAR). For those fortunate enough to receive ART, HIV/AIDS has become a chronic lifelong disease, one that requires strict adherence to treatment. Well-functioning health systems are needed to ensure the appropriate treatment of chronic diseases and achieve long-term retention in care, including regular follow-up appointments, patient education, adherence counselling, defaulter tracing and data monitoring. However, in health systems of many countries in the sub-Saharan Africa, such services are weak, as these health systems were primarily designed to deal with mother and child health issues and acute infections. Consequently, the current health systems typically lack the skills and capacity to manage chronic lifelong illness [1].

One of the major challenges in high HIV prevalence countries with weak health systems is the retention of the increasing numbers of patients on lifelong ART. Health systems in these countries need a fundamental overhaul of service delivery platforms if patients with HIV and other chronic illnesses are to be appropriately managed [2].

The impact of new additional financing for HIV programmes on health systems and non-HIV-related health services has been discussed extensively in recent years. Some assert that new additional financing, while enabling the scale up of HIV services, has overburdened already weak health systems, and thus worsened access for the target population [3,4]. In contrast, others indicate that HIV programmes and funding may also have a positive impact on health systems by reinforcing health support functions, such as human resources, infrastructure, laboratories and drug supply [5-7]. Yet, few studies have systematically explored the possible positive spill-over effects of HIV/AIDS programmes on health systems [8].

This study analyzes the effect of ART scale-up interventions on: (i) human resources; (ii) non-HIV service delivery platforms; and (iii) non-HIV health outcomes and goals, mainly focusing on primary health and maternal and child care indicators. The study draws on evidence from Malawi and Ethiopia: these countries, both confronted with a high number of people in need of ART, have successfully scaled up ART services in recent years, in spite of initially weak health systems. The study focuses on the impact of HIV programmes and funds on human resources for health and subsequently on health outcomes, using an impact pathway derived from a health systems conceptual framework by van Olmen *et al*[9].

Malawi and Ethiopia are low-income countries with, respectively, populations of 12.6 million and 73 million, and estimated HIV prevalence of 14.4% and 4.6% (Table 1); each country had more than a million people living with HIV in 2004 at the start of the HIV programme expansion. In spite of domestic and international efforts, health systems in these countries remain weak and underfunded. Hence, there are chronic shortages of human resources, infrastructure and essential drugs. Malawi and Ethiopia have some of the lowest ratios of medical doctors per 100,000 people in the world as a result of low training output, a relatively high brain drain and the severe adverse impact of HIV on the health workforce [13].

**Table 1:** Malawi and Ethiopia: Key population, economic and HIV-related indicators, 2004

	**Malawi**[**11**]	**Ethiopia**[**12**]
**General**[**10**]		
Total population	12.6 million	73 million
GDP (US$) per capita	208	138
Human Development Index (rank)	0.400 (166)	0.371 (170)
Total health expenditure (US$) per capita	38	19
% GDP	6.1	4.3
Life expectancy at birth (years)	37.5	48.8
**HIV-related indicators**		
HIV prevalence	14.4%	4.6%
Total people living with HIV/AIDS	1,100,000	1,200,000
People in need of ART – CD4 <200 cells/mm^3^	170,000	242,543
ART provided	13,183	9,000
Active and alive on ART	198,864	176,632
% ART coverage – CD4 <200 cells/mm^3^	6%	1.6%

ART, antiretroviral treatment; GDP, gross domestic product.

To scale up ART, both countries adopted a community-focused public health model. ART care activities were gradually decentralized to peripheral health centres and integrated into primary healthcare, which implied simplification, standardization and rationalization of treatment protocols. This was accompanied by a shift of several clinical and administrative tasks to lower-level health cadres and the gradual involvement of the community in ART care delivery [14-16].

In 2004, the HIV epidemic in Malawi highlighted the crisis of human resources for health, and triggered the introduction of an “Emergency Human Resources Program”. This human resources plan was funded by several donors, including the United Kingdom’s Department for International Development, and targeted financing for HIV from the Global Fund [17]. Training, incentives and salary top ups were used to recruit and retain health staff [18]. Pre-service training capacity for professional health workers was boosted and medical doctors were imported. The role of health surveillance assistants, until then mainly in charge of minimum basic primary healthcare activities, was strengthened. Together with newly created cadres, patient support attendants or expert patients, they now formed a network in the community for treatment adherence support, drug refill and defaulter tracing of patients not showing up for appointments. Regular refreshment training and supervision activities were introduced to ensure consistency and quality of care. Additionally, efforts were made to facilitate access to ART care for health staff [17].

Similarly, in 2003, the Ethiopian Federal Ministry of Health launched a new healthcare plan, the “Accelerated Expansion of Primary Health Care Coverage”, to extend quality preventive and selected curative healthcare services to the entire population, with special attention to mothers and children and people living in remote areas. Health extension workers were trained and appointed to each village, and were from now, in charge of 16 packages of basic preventive and selective curative services [19]. As the HIV epidemic worsened, in 2004, the national HIV/ AIDS policy was adapted in line with the existing health extension programme, and health extension workers were appointed for additional HIV-related tasks.

By then, the human resources crisis was recognized as a major bottleneck, and a comprehensive strategy for human resources for health was thus developed with the support of the World Bank, the Global Fund and PEPFAR [20-23]. This plan focused on the recruitment and retention of health workers through pre-service training of health staff , introducing incentives packages, shifting tasks to lower-level health cadres, and creating new cadres beyond the health extension workers. Community counsellors, HIV-positive peer educators and home-based care providers were appointed to health services, and were responsible for monitoring and evaluation, ART administration, adherence counselling and defaulter tracing. They link the community interventions to the formal health services. Concurrently, a significant proportion of the HIV-targeted resources was invested in additional health system strengthening interventions, such as the construction and revamping of health infrastructures and laboratories [20,21].

## Methods

**Figure 1:**
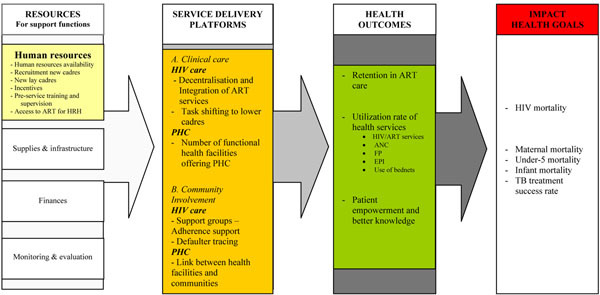
**Impact pathway of adapted human resources policies and ART retention support interventions on health outcomes and goals.** ART, antiretroviral therapy; HRH, human resources for health; PHC, primary health care; ANC, antenatal care; FP, family planning; EPI, expanded programme on immunization; TB, tuberculosis.

**Figure 2:**
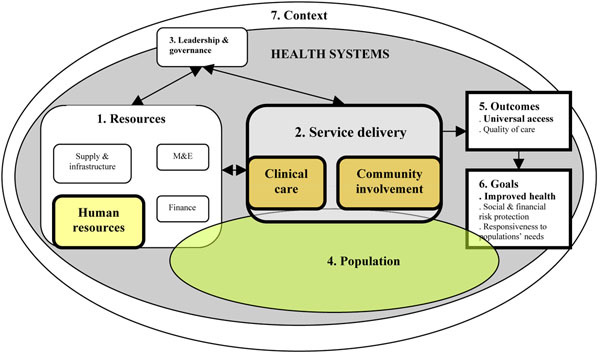
Conceptual framework health system, adapted from van Olmen *et al*. [9,24].

The pathway displayed in Figure 1 was used to examine the effect of ART scale-up interventions on health resources, health service delivery platforms and, subsequently, on general health outcomes and goals. This pathway draws upon a health system framework from van Olmen *et al* (Figure 2) [9,24], which links the allocation and management of resources (human resources, finances, infrastructure and supplies, and monitoring and evaluation) (Figure 2: 1) to the performance of the service delivery platforms (Figure 2: 2). All these functions require good governance and strong leadership (Figure 2: 3) taking into account the needs and demands of the population (Figure 2: 4), to reach the overall health outcomes (Figure 2: 5) and goals (Figure 2: 6) Finally, as the health system is embedded in a broader context, several internal and external factors can influence the final health outcomes and goals (Figure 2: 7).

This article focuses mainly on the possible positive spill-over effect of ART scale-up interventions on human resources policy changes and the health workforce. The principal indicators examined are: health staff availability (Table 2); the number of newly recruited staff; the number and type of new lay cadres; and the pre-service training and supervision offered.

**Table 2:** Number of health workers per 100,000 populations – Malawi (2004-2009) and Ethiopia (2002-2009)

	**Malawi**[**17**]		**Ethiopia**[**21**]	
	
	2004	2009	2002	2009
**Total number of health workers^§^**	**87**	**144**	**30.7**	**92.7**
Number of medical doctors	1.1	2	2.8	2.8
Number of nurses	25	37	17.8	20.4
Community health worker/Health surveillance assistant/Health extension workers	41	80	4.2	41

^§^The total number of health workers is composed of the number of medical doctors, nurses, community health workers and other health worker cadres (nurse assistants, clinical officers, medical officers, lab technicians, etc.), which are not specified in Table 2.

Second, we looked at the impact of health workforce changes on service delivery platforms. We considered both clinical care and community involvement, including ART care delivery. Clinical ART care is defined as the delivery of integrated and decentralized ART services at health facility level. Community involvement in ART care relates to tasks performed in or by the community, such as adherence support groups and defaulter tracing. Main indicators are the number of functional health facilities and the number of health facilities available for offering primary healthcare. Subsequently, we analyzed the effects of adapted service delivery platforms on HIV- and non-HIV-related health outcomes and goals.

The main indicators considered for health outcomes and goals are, respectively: retention in care; the utilization rate of primary health and maternal and child care services; the maternal, infant and under-five mortality rate; and the TB treatment success rate, as the maternal and child health and tuberculosis services display the most overlap with HIV activities. The impact of the other health system resources, i.e., drug supply and monitoring and evaluation, was not analyzed, as both countries implemented parallel systems for antiretroviral procurement and supply and for HIV monitoring and evaluation [21,25].

Key health and health services data on the indicators just described were compiled and compared using several sources: ministry of health documents (national health reports and working plans, and donor documents), evaluation and activity reports, scientific publications on human resources and health systems, and contacts with key informants in the countries. These data were compared with health indicators by year using the WHOSIS database. The data retrieved for Malawi and Ethiopia pertains to 2004-2009 and 2005-2009, respectively.

## Results

### Human resources

The change in human resources policies in Malawi and Ethiopia led to an increase in the number of trained health staff at health facility and community level, respectively, by 65% and 302% (Table 2). In Malawi, the reinforcement of pre-service training resulted in a 72% and 12% increase, respectively, in the number of graduated medical doctors and nurses [17]. However, the major increase in the health workforce was noted among community health workers and lay workers. In Malawi, between 2004 and 2009, the number of trained health surveillance assistants increased significantly from 4000 to more than 10,000, and the proportion of health facilities with sufficient staffing available increased from 4% to 13% [17].

In Ethiopia, between 2005 and 2010, almost 34,000 health extension workers and 5500 community HIV lay counsellors and peer educators were trained and employed throughout the country. An additional 3573 health officers were trained as mid-level health professionals in charge of primary healthcare services, including ART care, and supervision of health extension workers. In a study in Rwanda, this task shifting to lower-level health cadres led to a 76% reduction of doctors’ time, freeing up more time for primary healthcare services [26]. Over the same period, the proportion of health workers receiving regular supervision increased by 27% [21].

### Service delivery

In both countries, the expansion of the health workforce facilitated the implementation of a public health approach and the progressive decentralization of ART care to the primary healthcare level. Medical and administrative tasks were delegated to lower-level health cadres. Nurses were involved in the initiation of ART and community health workers in adherence support, defaulter tracing, ART distributions and non-HIV-related tasks, such as vaccination, tuberculosis treatment, family planning and antenatal care.

At the same time, this health workforce expansion contributed to an overall increase in functional health facilities. In Malawi, between 2004 and 2010, the proportion of all health facilities providing immunization and family planning services increased steeply, from 9% to 74%. The availability of basic emergency obstetric services in health facilities rose from 2% to 65% [25]. In Ethiopia, maternal and child services (antenatal care, obstetrics, postnatal care and expanded programme on immunitization) were progressively decentralized, along with the HIV/AIDS services to the primary healthcare level. The number of health facilities providing integrated management of childhood illnesses services increased from 303 in 2007 to 1011 in 2009. Additionally, HIV funds were used to construct and revamp health facilities, increasing their number from 3544 in 2004 to 17,300 in 2010 [20].

### Health outcomes

**Figure 3:**
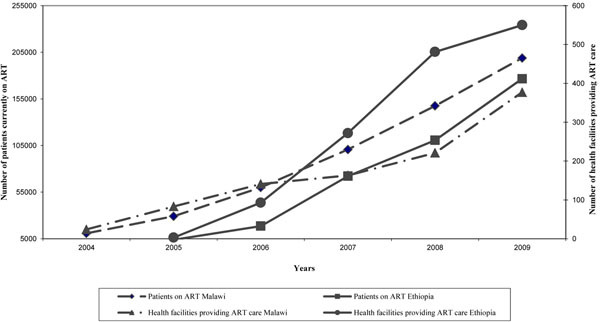
Number of patients currently on ART and number of health facilities providing ART care in Malawi and Ethiopia, 2004-2009.

The decentralization and integration of ART services in primary healthcare services contributed to a significant scale up of the number of patients on ART in Malawi and Ethiopia. By the end of 2009, respectively, 198,846 and 176,632 HIV-infected patients were on ART [27,28] (Figure 3).

In Malawi, the proportion of patients initiated on ART in WHO stage 4 decreased from 25% in 2005 to 9% in 2010. The 12-month retention rate had increased to 79% by 2009 [27,29](Table 3).

**Table 3:** Malawi and Ethiopia: Key population, economic and HIV-related indicators, 2009

	**Malawi**[**27**]	**Ethiopia**[**29**]
**General**[**10**]		
Total population	15 million	80 million
GDP (US$) per capita	354	364
Human Development Index (rank)	0.385 (153)	0.328 (157)
Total health expenditure (US$) per capita	62	26
% GDP	12.9	3.9
Life expectancy at birth (years)	50.9	55.8
**HIV-related indicators**		
HIV prevalence	12%	2.4%
Total people living with HIV/AIDS	1,100,000	1,200,000
People in ART need – CD4< 200	305,805	397,818
ART provided	271,105	241,759
Active and alive on ART	198,864	176,632
% ART coverage – CD4 < 200	65%	44%
% Children on ART	7.5%	5%
12- month Retention on ART	79%	72%

ART, antiretroviral treatment; GDP, gross domestic product.

During the same period, a remarkable improvement in the utilization of key primary healthcare services, including mother and child services, was noted [30] (Table 4). The proportion of children under five years with acute respiratory infections who sought treatment doubled. The proportion of assisted deliveries, antenatal care attendance and measles vaccination coverage also improved. The increased health workforce and the higher number of available health facilities contributed to a doubling of the primary health capacity in both countries. In addition, the increase in health service utilization rate can be attributed both to the choice to deliver HIV services at primary healthcare level and to the reinforcement of the links between HIV and maternal and child health services.

**Table 4:** User rate of primary healthcare services, Malawi (2004-2009) and Ethiopia (2005-2009)

	**Malawi**[**25**]		**Ethiopia**[**20**,**21**]	
	
Utilization of primary healthcare facilities	2004	2009	2005	2009
**Curative care**				
OPD annual visits per 1,000 population per year	800	1,290	300	330
Potential PHC capacity	NA	↑ of 49%	45%	90%
% children under-5 with ARI S/ or fever, treatment was sought	19.6%	51.8%	15.8%	39.1%
TB case detection rate	50.3%	49.7%	41.9%	47.5%
**Mother & child health**				
ANC attendance	NA	93%	46%	68%
% of birth deliveries in health facilities	38%	52%	15%	34%
Use of contraceptive methods	28.4%	41%^σ^	21.5%	33.6%
Measles vaccination coverage	59%	89%	55%	66%
% children & women using impregnated bed nets for prevention of malaria	13%	25.6%	42%	60%

ANC, antenatal care; ARI, acute respiratory infection; PHC, primary healthcare; OPD, out patient department.

The involvement of the community in care delivery encouraged social mobilization and improved access to health education. In Ethiopia, pregnant women in communities with an active health extension programme show up significantly earlier in pregnancy for antenatal consultations compared with those living in villages without health extension workers [20]. A study in a rural district in Malawi showed an eight-fold increase in the annual tuberculosis detection rate after involving community workers in the active case finding of tuberculosis, and a significant reduction in stigma in the community [31].

### The impact on health goals

In Malawi, the early mortality rate after ART initiation dropped from 15% (Quarter 3, 2005) to less than 5% (Quarter 2, 2010) [27]. In Addis Ababa, Ethiopia, a steep decline in population-level AIDS mortality was also reported following the introduction of ART [32].

In both countries, improvements in tuberculosis treatment success rates and declines in maternal, under-five and infant mortality rates can be potentially attributed to enhanced accessibility and use of health services (Table 5). A study analyzing the all-cause mortality rate in rural Malawi showed an overall reduction in mortality by 37% over an eight-year period (2000-2008) [33].

**Table 5:** Health goals in Malawi (2004-2009) and Ethiopia (2005-2009)

	**Malawi**[**25**]		**Ethiopia**[**20**]	
	
Utilization of primary healthcare facilities	2004	2009	2005	2009
**Mortality**				
Maternal mortality per 100,000 live births	1662	1140	937	590
Under-five mortality per 1000 live births	133	100	204‡	101
Infant mortality per 1000 live births	76	65	127	109
**TB treatment success rate**	71%	84.7%	79.3%	84%

^‡^ Data 1996/97.

## Discussion

The Malawi and Ethiopia country studies demonstrate how accelerated ART scale-up interventions triggered human resource policy changes, which contributed to increased human resource availability at primary care level, facilitating increased access and quality of primary healthcare and inducing a positive spill-over effect on general health outcomes and goals.

In both countries, the shortage of human resources for health was identified as a major bottleneck in ART scale up and was given high priority at all levels, with the adaption and boosting of human resources policies. Government and donors negotiated and agreed to use a substantial proportion of the available increase in HIV-earmarked donor funding in this period for general health system strengthening; it was obvious that public health ART care delivery could only be sustained through a reinforced health system. Within health system strengthening, HIV-earmarked funds were mainly used for financing human resources plans, which helped increase the health workforce availability, through better salaries, pre-service training, improved geographical distribution of health staff , and reinforcement of the community-based cadres [34]. In Ethiopia, HIV funds also addressed the lack of peripheral health facilities by constructing additional health facilities [20].

The creation and recruitment of new health cadres, in combination with task shifting of medical and administrative responsibilities to lower-level health cadres, catalyzed training and involvement of lower-level cadres in other non-HIV activities. In Malawi, access by health staff to ART significantly reduced absenteeism rates. ART saved at least 25% of health staff lives after 12 months of treatment [35,36]. In both countries, the increased motivation through regular incentives, training and supervision, and improved working conditions led to a better retention of existing health workers.

Collectively, these modifications contributed to an increase in the health workforce and the number of functional health facilities, which allowed the rapid scale up of ART services and the better retention of patients in ART care. Yet, this increase also facilitated the accessibility and coverage of non-HIV health services [37].

One of the major future operational challenges remains developing a model of care able to cope with the evergrowing case load of HIV-positive patients requiring lifelong treatment and retention in care adapted to the resources available. Further, incorporation of this model in the general health system and strengthening of the health system to ensure sustainability and scalability in the future needs to be addressed. There is a potential risk of saturating the existing health facilities, jeopardizing the quality of care. In order to deal with this challenge of achieving effective and sustainable ART care at primary healthcare level, community involvement, promotion of awareness and patient self-management are crucial [38-40].

Studies in Malawi, Ethiopia and South Africa have documented that the active participation of people living with HIV/AIDS and the community in the planning and provision of care not only facilitated adherence to lifelong ART, but also led to increased patient treatment literacy, empowerment and stronger linkages with the community [41-43]. This patient empowerment potentially gives more voice to patients to advocate and claim their rights to healthcare. This will, in turn, have a positive effect on the uptake of health services. Although the response to the HIV epidemics in Malawi and Ethiopia was exceptional and not representative for other countries with high HIV prevalence, some lessons learnt on how scaling up ART offers an opportunity to strengthen the health system can be useful in other contexts.

This analysis points to the possibility of obtaining improved health outcomes beyond HIV through scaled-up ART interventions if this process is planned with a comprehensive and long-term vision, involving all actors, especially at community level. The country examples show that a human resources analysis with a system-wide view is crucial from the very beginning, when one considers the implementation or adaptation of disease-specific activities. The different bottlenecks of the health system and possible spill-over effects should ideally already be identified and addressed proactively when planning an ART programme expansion, as was done for the human resources issues in Ethiopia and Malawi [44,45].

In addition, it is important to improve coherence and coordination between the different chronic disease responses, primary healthcare and health system strengthening efforts to avoid fragmentation, improve the interface, and adapt policies to maximize synergies. From these case studies, we also learn that to achieve better synergies between HIV disease-specific programmes and health systems, several preconditions need to be taken into account, including strong political commitment from governments and decision markers, donor flexibility and sufficient financial resources to effectively execute planned activities.

There are a number of limitations to our analysis. First, the analysis links the positive spill-over effect of changes in human resources policy due to funding from global health initiatives to health outcomes. Our results, however, cannot demonstrate a direct link of the human resources policy changes on non-HIV health outcomes due to data limitations and, more specifically, the availability of a limited number of indicators measured at two points in time only. To measure and control the impact of HIV funds on non-HIV outcomes, a larger set of health indicators with more measurements over time would be needed.

Second, as health system strengthening is a complex endeavour, with a variety of funding sources, it is difficult, indeed, to attribute overall results to one specific intervention. Other internal or external contextual factors, such as a better socio-economic situation, a more stable political situation, a higher level of education and an improved nutrition status, might confound these results as they can obviously also boost the general health status of the population. Moreover, efforts of other disease-specific programmes, free access to healthcare and improvement of primary healthcare services, including maternal and child healthcare, can also lead to better health outcomes. However, our analysis indicates no documented evidence of substantial changes in other factors, programmes or patient user fees for health services in this time period.

Third, another limitation in our study is the use of routine data for this analysis, which might be incomplete, including possible recording errors or biases. We did, however, cross check data from different sources and found no major inconsistencies.

More systematic quantitative and qualitative research in several countries is definitely required to evaluate the impact of the different ART programme components on health systems and to assess how ART delivery models can contribute to health system strengthening. There is a need to explore the potential use of HIV resources and adapted ART care delivery platforms in the care of non-HIV diseases and the role and the impact of increased user involvement on general health outcomes.

Most developing countries are experiencing an increased burden of non-HIV chronic diseases, such as diabetes and hypertension. This burden remains, however, largely neglected in terms of service delivery. Scaling up the ART service model, based on integration in primary healthcare, self-management and community involvement, could have a potential role in the management of other chronic diseases requiring continuity of care [46-48].

## Conclusions

New additional financing has been invested by global health initiatives for health system strengthening to scale up HIV programmes in sub-Saharan African countries, inspired by a growing awareness that these systems are critical to offer long-term sustainable management of HIV. Our study shows that the scale up of ART services does not adversely affect the performance of non-HIV health services. On the contrary, our study shows that the health outcomes of several non-HIV services improved in Malawi and Ethiopia during the same period. Based on both country examples, we conclude that interventions aimed at the expansion of ART services and improvement of long-term retention of patients in ART care can have positive spill-over benefits on the general health system.

In both countries, HIV/AIDS-earmarked resources were used to strengthen the health system through tackling the shortage of human resources for health. Increased access to health facilities at community level due to decentralization of services and task shifting to lower-level health cadres and increased involvement of community health workers through community-based networks may have contributed to improved health outcomes beyond HIV.

The ART service delivery models can thus potentially serve as an effective *modus operandi* to tackle other common chronic diseases in low-income countries. These models allow countries with relatively weak health systems to cope with the ever increasing number of patients requiring care, to reduce the workload in the health facilities, and to improve retention in care. For this to happen, the different health partners need to acknowledge and capitalize on these interactive effects when planning and implementing health activities.

## Competing interests

The authors declare that they have no conflicts of interest.

## Authors’ contributions

FR conceptualized the study and wrote a first draft, which was edited by all authors. YA and ES assisted with country-specific data collection. MP, MPP and WVD checked scientific soundness and reviewed the manuscript several times. All authors (WVD, FR, MP, MPP, RA, EW, BC, EJS and YA) contributed to the intellectual content of this article. FR and MP finalized the manuscript. All authors read and approved the final version prior to publication.
